# Effects of temperature variability and extremes on spring phenology across the contiguous United States from 1982 to 2016

**DOI:** 10.1038/s41598-020-74804-4

**Published:** 2020-10-21

**Authors:** Lingling Liu, Xiaoyang Zhang

**Affiliations:** 1 Geospatial Sciences Center of Excellence (GSCE), South Dakota State University, 1021 Medary Ave, Brookings, SD 57007 USA; 2 Department of Geography & Geospatial Sciences, South Dakota State University, Brookings, SD 57007 USA

**Keywords:** Climate-change impacts, Phenology

## Abstract

Warming climate and its impact on vegetation phenological trends have been widely investigated. However, interannual variability in temperature is considerably large in recent decades, which is expected to trigger an increasing trend of variation in vegetation phenology. To explore the interannual phenological variation across the contiguous United States (CONUS), we first detected the onset of vegetation greenup using the time series of the daily two-band Enhanced Vegetation Index (EVI2) observed from the AVHRR Long-Term Data Record (1982–1999) and the MODIS Climate Modeling Grid (2000–2016). We then calculated the interannual variation in greenup onset during four decadal periods: 1982–1989, 1990–1999, 2000–2009 and 2010–2016. Further, the trend of interannual variation in greenup onset from 1982 to 2016 was analyzed at pixel and state levels. Extreme phenological events were also determined using a greenup onset anomaly for each pixel. Similar approaches were applied to spring temperatures to detect extreme years and to the temporal trend of interannual variation to explain the phenological variation. The results revealed that 62% of pixels show an increasing interannual variation in greenup onset, and in 44% of pixels, this variation could be explained by the temperature. Although extreme phenology occurred locally in different years, three nationwide extreme phenological years were distinguished. The extreme warm spring that occurred in 2012 resulted in the occurrence of greenup onset as much as 20 days earlier than normal in large parts of the CONUS. In contrast, greenup onset was much later (up to 30 days) in 1983 and 1996 due to cool spring temperatures. These findings suggest that interannual variation in spring phenology could be much stronger in the future in response to climate variation, which could have more significant impacts on terrestrial ecosystems than the regular long-term phenological trend.

## Introduction

Vegetation phenology is the expression of the seasonal cycles of plant processes and their responses to climate change, which can be used to document the effects of climate change on individual plant species^1,2^. Field-based phenological observations have been recorded beginning thousands of years ago in China^3^, in the 1700s in Europe, and in the 1800s in Japan. Recently, various networks of field phenological observations, such as the National Phenology Network (NPN) in the United States, the Pan European Phenology network, and PlantWatch in Canada, have been established. Historical phenological observations for specific species have been widely used to determine long-term trends and to explore the response of terrestrial ecosystems to climate change^4–6^.

Vegetation phenology at regional and global scales has been primarily derived using observations from satellites that could provide wall-to-wall observations^7,8^. Satellite-derived vegetation phenology is called land surface phenology (LSP), and it reflects the seasonal pattern of variation in vegetated land surfaces^9^. Based on long-term LSP data, numerous studies have focused on the detection of temporal trends in the start and end of the growing season, as well as the length of the growing season, during the last several decades^10–12^. Although the temporal trend of the start of the growing season (also known as greenup onset) generally ranged from 0.2–0.5 days/year in northern middle-high latitudes, the trend’s directions and magnitude were found to differ dramatically among various studies even for the same region. For example, Zhang et al.^13^ revealed earlier greenup onset in mid-high latitudes from 1982 to 2005, while Jeong et al.^14^ reported later greenup onset trends in large parts of North America from 1982 to 2008. In contrast, both Reed^15^ and White et al.^16^ indicated that there were no significant trends in greenup onset across almost all of North America. These controversial temporal trends from satellite detection are likely associated with uncertainties in both detecting phenological metrics and dealing with large interannual variations.

Trend analysis only reflects the overall pattern of gradual change or general tendencies in phenological metrics over a period, but long-term phenological dynamics often contain considerable interannual variation. The interannual variation or variability refers to the degrees of fluctuation of the phenological process in response to climatic variability^17^. The large interannual variation in vegetation phenology has been recently recognized in phenology and climate change communities. Specifically, Zhang et al.^18^ found that the interannual variation in greenup onset could be more than one month in precipitation-controlled tropical and dry climates globally, while it could be as much as half of a month in temperature-controlled temperate, cold, and polar climates. At a regional scale, such as New England, Melaas et al.^19^ revealed that the start and end of the growing season varied by as much as four weeks over the 30-year record of Landsat images. In this region, Friedl et al.^20^ showed that leaf emergence occurred up to two weeks earlier than normal in 2010 and 2012 because spring temperature in these two years was nearly 3 °C warmer than the long-term average temperature from 1971 to 2000. Interannual variation in phenology could also be impacted by other factors, such as snow cover in mountain and cold environments and precipitation in semiarid regions^21–23^. The large interannual variations in phenological events have significant impacts on the feedbacks of the seasonality of albedo, surface roughness length, canopy conductance, and fluxes of water, energy, and CO_2_^24–27^.

Extreme phenological events could occur in response to weather and climate extremes, which could have the greatest direct impacts on human health, agricultural development, and terrestrial ecosystems^28,29^. Spring flowering timing could be greatly advanced and alter pollen seasonality and concentration due to extremely warm temperatures, which could impact allergic diseases such as hay fever^30^. Moreover, an extremely early spring phenology with prolonged and increasing evapotranspiration could amplify summer heat waves and droughts ^31,32^. The feedback from extreme vegetation phenology was evident in the 2003 extreme heat wave in Europe^33,34^ and the 2012 extreme drought in the central United States^32^. Heat waves have important consequences for public health, such as the reductions in labor capacity^35^ and increases in heat-related mortality^36^. Extreme droughts significantly reduce crop yield^37^. In addition, vegetation species adapt to climate change through phenotypic plasticity or adaptive evolution, and extreme climate conditions may result in a potential increase in the risk of extinctions of local species^38^. Therefore, understanding interannual phenological variations and extreme phenological events in response to climate variation and extremes is of great significance for mitigating global challenges such as food insecurity, water scarcity, and biodiversity loss^17,39^.

Therefore, this study aims to explore the spatiotemporal changes of interannual variations and extremes in spring vegetation phenology across the CONtiguous United States (CONUS, the United States excluding Alaska and Hawaii). By analyzing spring phenology derived from the Advanced Very High Resolution Radiometer (AVHRR) data from 1982–1999 and the Moderate Resolution Imaging Spectroradiometer (MODIS) records from 2000 to 2016, this study attempts to answer the following questions: (1) what is the magnitude of interannual variations in spring phenology during the past several decades? (2) have interannual variations in spring phenology increased from 1982 to 2016? (3) are interannual variations in spring phenology driven by the variations in temperature? (4) in which years and areas did extreme phenological events occur over the past several decades?

## Materials and methodology

### Satellite data and land surface phenology detection

We collected the AVHRR LTDR (AVH09C1 version 4) from 1982 to 1999 and the MODIS CMG (MOD09CMG, Collection 6) from 2000 to 2016. The AVHRR LTDR was designed to produce a data set to be consistent with MODIS CMG records for land climate studies^18,40^. The combination of AVH09C1 and MOD09CMG provides the longest daily surface reflectance that covers the globe at a spatial resolution of 0.05° (~ 5 km at equator).

We calculated the daily two-band Enhanced Vegetation Index (EVI2) and Normalized Difference Vegetation Index (NDVI) using red and near-infrared reflectance in AVH09C1 and MOD09CMG in order to detect land surface phenology (LSP). Specifically, the EVI2 time series was used to extract LSP in this study as it has several advantages over NDVI: (1) EVI2 reduces the sensitivity to soil and atmospheric effects and remains sensitive to variation in canopy density where NDVI becomes saturated^41,42^, (2) EVI2 is more sensitive to vegetation gross primary production, whereas NDVI is more representative of the total leaf variation on a vegetation canopy^43–45^, and (3) EVI2 detects more accurate phenological metrics than NDVI in comparison with field observations, flux tower data, and PhenoCam retrievals^46–50^. Due to the fact that anomalously large EVI2 values could arise from spuriously low red band values caused by an inaccurate atmospheric correction or a variety of other factors, EVI2 values were identified and removed if they were larger than 90% of the corresponding NDVI values or larger than 110% of any EVI2 values generated during the previous and successive one-month periods^50^. Subsequently, the daily EVI2 and NDVI values were aggregated to 3-day composites using a maximum value composite (MVC) procedure to reduce cloud contamination initially and to reduce data volumes while retaining fine temporal resolution^51^.

Land surface phenology was extracted from the EVI2 time series for each pixel. This processing was performed following a previous study^51^. Briefly, we first identified the minimum snow-free EVI2 value for each pixel to remove the effect of snow. The snow information for MODIS data was provided in the quality assurance (QA) of MOD09CMG; for the AVHRR LTDR data, it was acquired from the NOAA (National Oceanic and Atmospheric Administration) global vegetation index product^51,52^. Subsequently, the fill values in the EVI2 time series caused by cloud cover and other factors were replaced using a moving average of two neighboring good quality values. Finally, the Savitzky-Golay filter was applied to attenuate irregular variations in the EVI2 time series after any cloud-contaminated values were replaced. Based on the smoothed EVI2 time series, a hybrid piecewise logistic model (HPLM) was applied to reconstruct EVI2 temporal trajectories. The curvature change rate derived from the HPLM was used to identify the phenological transition dates during a vegetation growing season^53,54^. In this study, we focused on greenup onset, which was thought to exert a major effect on the carbon balance of terrestrial ecosystems^55^. In addition, we calibrated AVHRR-based spring phenology to minimize the potentially possible systematic differences between AVHRR-based and MODIS-based phenological time series using linear regression models^10^. Specifically, greenup onset at the pixel level in 1999 and 2000 was first predicted using linear models established based on the AVHRR-based (1982–1999) and the MODIS-based (2000–2016) time series. The difference between the two model predictions in 1999 and 2000 was then applied to adjust greenup onset from the AVHRR^56^.

### Temperature data

Temperature is the most important trigger of spring phenology^57^. We downloaded the North America Regional Reanalysis (NARR) data set for the period of 1982–2016 from the website (https://nomads.ncdc.noaa.gov). The NARR was developed at the Environmental Modeling Center (EMC) of the National Centers for Environmental Prediction (NCEP), and it contains 3-hourly temperature at a spatial resolution of 32 km with the Lambert conformal conic projection^58,59^. To spatially match the land surface phenology data, we converted the Lambert projection to a latitude/longitude geographic projection (0.25°) and subsequently resampled to 0.05° using the nearest neighbor algorithm.

### Investigation of interannual variation in greenup onset

The interannual variations in greenup onset were quantified using standard deviation (STD), which was calculated using the following formulas:

1 $$\overline{LSP} = \frac{{\sum\nolimits_{i = 1}^{n} {LSP_{i} } }}{n}$$

2 $$STD = \sqrt {\frac{{\sum\nolimits_{i = 1}^{n} {(LSP_{i} - \overline{LSP} } )^{2} }}{n - 1}}$$

where $LSP_{i}$ is the greenup onset for a certain year *i*, *n* is the number of years, $\overline{LSP}$ is the mean value of greenup onset for *n* years, and STD is the standard deviation of greenup onset within *n* years.

Specifically, we applied two methods to investigate the temporal and spatial patterns of interannual variation in greenup onset. First, we calculated the STD of greenup onset during the four decadal periods of 1982–1989, 1990–1999, 2000–2009, and 2010–2016 in order to provide the overall difference in interannual variations at a decadal scale. Second, we calculated the STD for a short continuous period using a moving window of 5 years, otherwise known as the five-year centered moving STD. The moving STD generated a time series of 31 data points in each pixel, which was suitable to represent short-term interannual variation. The slope of the moving STD against the year was calculated using the Theil–Sen estimator to describe the temporal trend in interannual variation. The significance level of the temporal trend was determined using the Mann–Kendall test. We also calculated the percentage of pixels with increasing and decreasing interannual variation in greenup onset for each state and the entire CONUS, respectively, which could provide valuable information within political regions for national climate assessment^60^.

We further detected the years of extreme phenological events that had strong contributions to interannual variation in greenup onset across the entire CONUS. The extreme years were determined using the anomalies, which are commonly used to describe the deviation of vegetation properties in an individual year from a long-term mean^61,62^. The anomaly in greenup onset was calculated using the following formula:

3 $$LSP_{i}^{\prime } = LSP_{i} - \overline{LSP}$$

where $LSP_{i}$ is the greenup onset for a given year *i*, $\overline{LSP}$ is the mean value of greenup onset from 1982 to 2016, calculated using Eq. (1) with n = 35, and $LSP_{i}^{^{\prime}}$ is the anomaly of greenup onset for a given year *i*.

The extreme years were then detected when the absolute value of the anomaly for a given year was larger than two standard deviations for each pixel, where the standard deviation was calculated, using the greenup onset from 1982 to 2016, with Eq. (2).

### Investigation of the responses of interannual variation in greenup onset to temperature variation

In parallel to greenup onset, using the Theil–Sen estimator and Mann–Kendall test, we determined the temporal trend and extreme years of interannual variations in spring temperature, because spring temperature is the major driver of spring phenology. The spring temperature used in this study was defined as the mean temperature within a specified period length (PL, days) prior to the occurrence of the phenological event^63–65^. This selection was based on the fact that the temperature prior to greenup onset is the most important driver of greenup onset^20^ and the timing of greenup onset varies greatly across the CONUS. Thus, the time period of temperature controlling spring phenology in each pixel varies instead of being a fixed period or specific months. In order to determine the optimal PL for a given pixel, we first calculated the correlation coefficient between the time series of greenup onset (from 1982 to 2016) and the time series of mean temperature (from 1982 to 2016) during a period prior to greenup onset. With this period, which varies from 1 to 90 days prior to greenup onset with an interval of 1 day, a set of correlation coefficients were obtained. The period with the largest correlation coefficient was selected as the optimal PL for a given pixel. The pixel-based optimal PL was then applied to each year from 1982 to 2016 to calculate the mean temperature in spring. Further, we calculated the percentage of pixels with increasing and decreasing interannual variations in temperature across the entire CONUS to compare with variations in greenup onset. In addition, the percentage of pixels with consistent increasing or decreasing interannual variations in greenup onset and temperature was computed across the entire CONUS and all individual states. Finally, extreme temperature years were detected using the same method that was used for the detection of extreme greenup onset occurrences.

## Results

### Interannual variation in greenup onset during different decades

**Figure 1:**
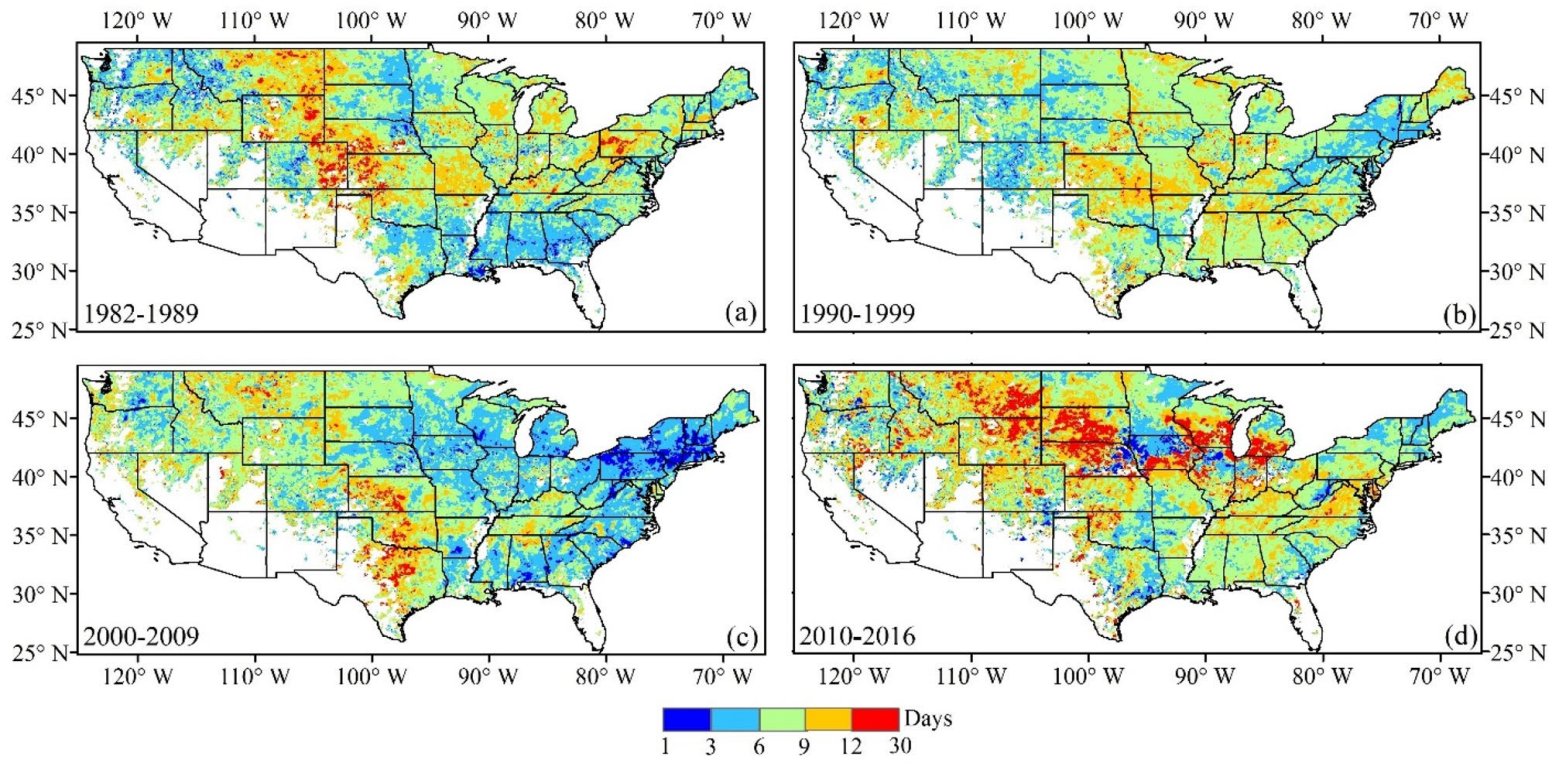
Spatial pattern of interannual variations in greenup onset during four different periods: (**a**) 1982–1989, (**b**) 1990–1999, (**c**) 2000–2009, and (**d**) 2010–2016. The white color represents water or pixels where vegetation greenup was not continuously detected in spring during the period of 1982–2016.

Figure 1 presents the interannual variation in greenup onset within each decade from 1982 to 2016. During the period of 1982–1989, the interannual variation (STD) was smaller, with values lower than 6 days in northwestern and southeastern regions (Washington, western Montana, northern Idaho, Georgia, Alabama, and Mississippi). In contrast, the largest variation (STD > 12 days) appeared in the Midwest region (eastern Montana and Colorado, Wyoming, western Nebraska, and Kansas) (Fig. 1a). During the period 1990–1999, the interannual variation was 3–12 days in most states. The central CONUS showed stronger interannual variation than the eastern and western areas (Fig. 1b). In the following decade (2000–2009), the variation differed substantially in the eastern and western CONUS. The variation was less than 6 days in most states in the eastern CONUS (New York, Pennsylvania, Massachusetts, and Vermont), while it was larger than 6 days in most states in the central and western CONUS, where the largest variation appeared in Kansas, Oklahoma, and Texas (Fig. 1c). In recent years (2010–2016), interannual variation in greenup onset was larger than it was for the previous several decades. The variation was larger than 12 days in most parts of the Midwest (such as South Dakota, Montana, Wyoming, Nebraska, Wisconsin, and Michigan) (Fig. 1d). Overall, the interannual variation was largest during the period of 2010–2016, although the spatial pattern of variation differed greatly in each decadal period.

**Figure 2:**
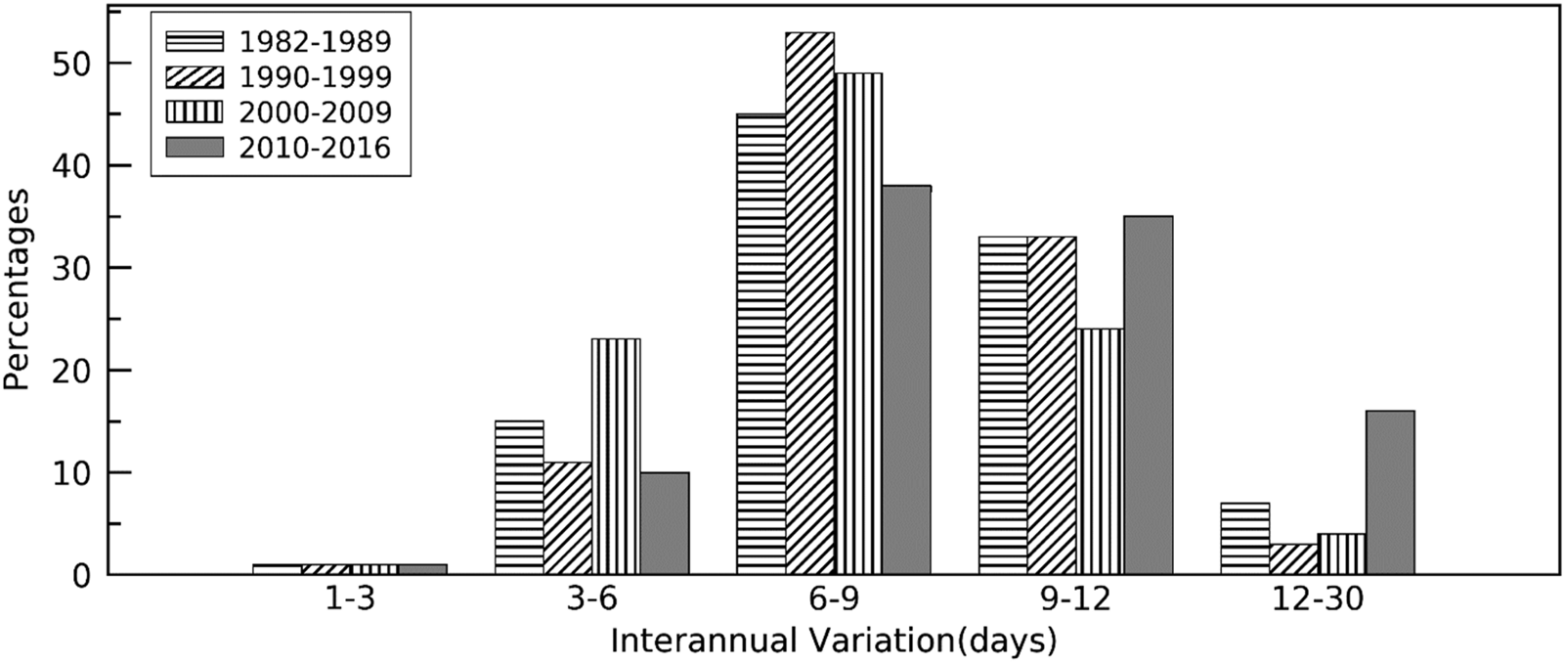
A histogram of areas (percentage of total pixels with continuous long-term phenology detections in spring across the CONUS) with different variation levels during four different decades.

Figure 2 shows the percentage of pixels with different interannual variation levels across the CONUS for each decade. The interannual variation in greenup onset mainly ranged from 6–12 days, which accounted for 78%, 86%, 73%, and 73% of the pixels during the periods of 1982–1989, 1990–1999, 2000–2009, and 2010–2016, respectively. The variation was distinctively larger during the period of 2010–2016 compared to other periods. The percentage of pixels with a variation larger than 9 days was 51% during the period of 2010–2016, compared to 40%, 36%, and 28% during the periods of 1982–1989, 1990–1999, and 2000–2009, respectively (Fig. 2).

### Temporal trends of interannual variation in greenup onset and temperature

**Figure 3:**
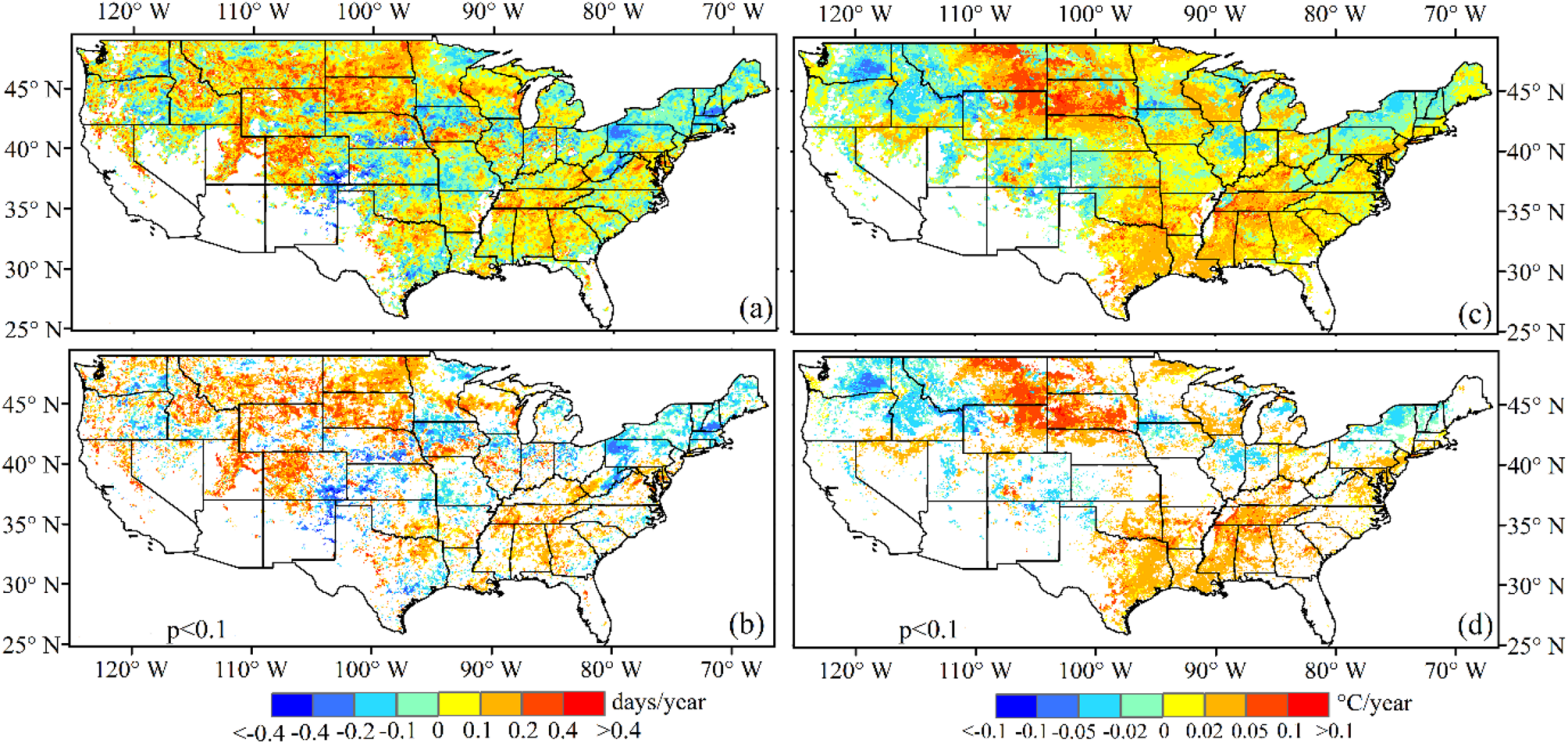
Spatial pattern of temporal trends in interannual variations (STD within a moving window of 5 years) in (**a**, **b**) greenup onset and (**c**, **d**) spring temperature from 1982 to 2016. (**b**, **d**) Present the pixels with a significance level of less than 0.1. The white color represents water or pixels where greenup onset was not continuously detected or was without significant trends during 1982–2016.

**Figure 4:**
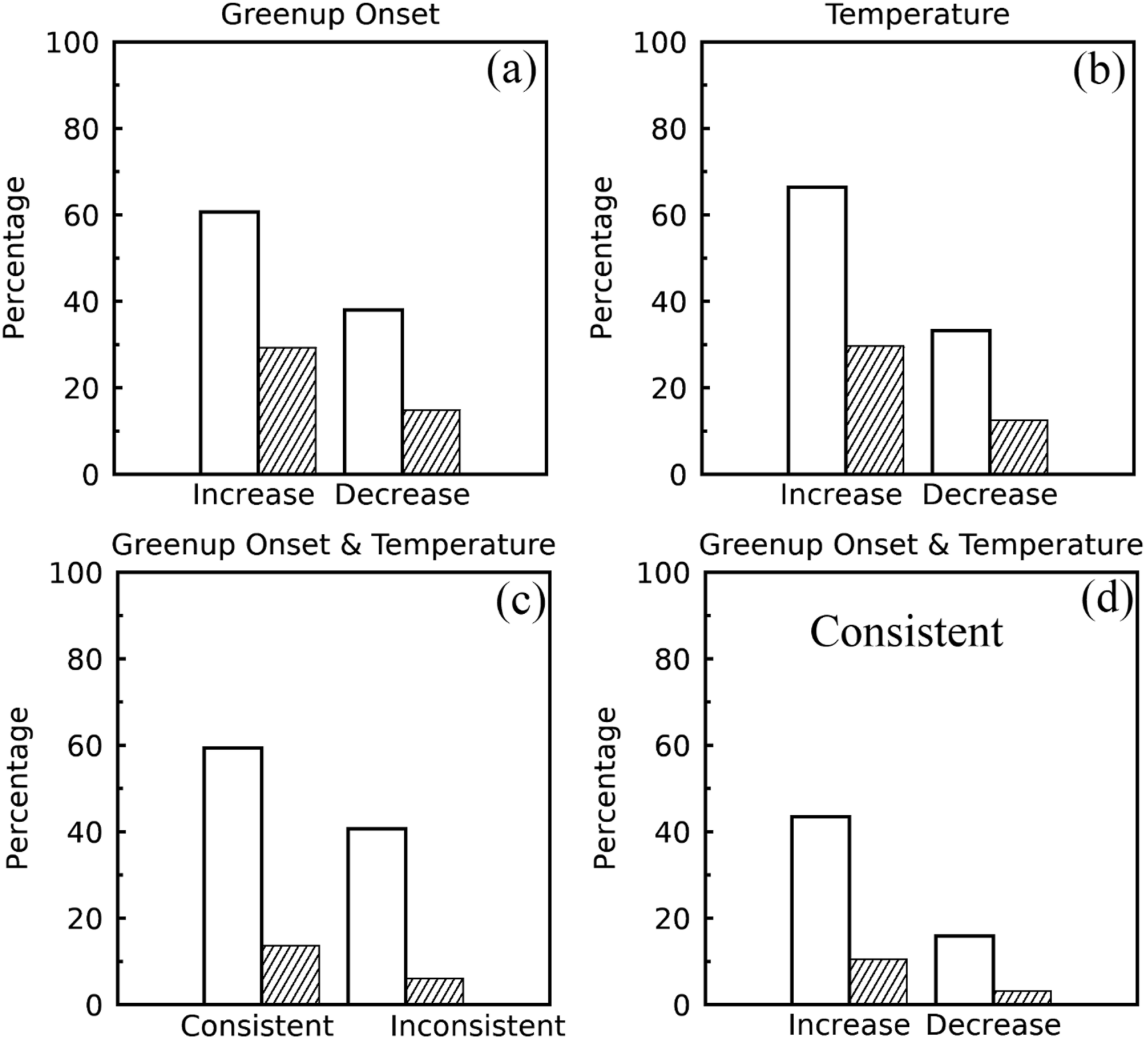
Pixel percentages with increasing and decreasing trends of interannual variation from 1982 to 2016 across the CONUS. (**a**) Greenup onset, (**b**) spring temperature, (**c**) trends of greenup onset that are separately consistent and inconsistent with temperature trends, (**d**) consistently increasing and decreasing trends in interannual variation between greenup onset and temperature. The patterned boxes represent the pixel percentage with the trends at a significance level less than 0.1, while the white boxes indicate the pixel percentage for both significant and non-significant trends.

Figure 3a,b show the spatial pattern of the temporal trend in interannual variation representing the five-year centered moving standard deviations of greenup onset from 1982 to 2016. An increasing trend in interannual variation appeared in most regions (Fig. 3a), especially in northwestern and southeastern regions (such as South Dakota, North Dakota, Montana, Wyoming, Colorado, Utah, Wisconsin, Alabama, Georgia, and Tennessee). The decreasing trend only occurred in a few scattered regions (such as southeastern Washington, eastern Oregon, southern Nebraska, Pennsylvania, New York, New Hampshire, and Maine). Statistically, 62% of pixels exhibited the increasing trend, 29% of which were significant (P < 0.1, Fig. 4a). In contrast, 38% of pixels showed decreasing trends, 15% of which were significant (P < 0.1, Fig. 4a).

Similar to greenup onset, spring temperature also indicated a spatial pattern in both increasing and decreasing trends in interannual variation from 1982 to 2016 (Fig. 3c,d). Overall, 67% of pixels revealed an increasing interannual variation in temperature (30% of which had P < 0.1, Fig. 4b). The remaining 33% of pixels exhibited a decreasing temperature trend (12.5% of which had P < 0.1, Fig. 4b).

Figure 4c,d present the consistency of the temporal trend of interannual variation between greenup onset and spring temperature. The trend of interannual variation in spring temperature was consistent with that in greenup onset in 60% of pixels across the entire CONUS (Fig. 4c). Among these pixels, both greenup onset and spring temperature had consistent increasing trends in 44% of pixels and consistent decreasing trends in 16% of pixels (Fig. 4d).

However, for 41% of pixels, the direction of the temporal trend in interannual variation in greenup onset was opposite to that in spring temperature; 6% of these pixels exhibited a significant trend (P < 0.1, Fig. 4c). These contrasting temporal trends mainly occurred in states in the Rocky Mountain area (such as Idaho, Wyoming, Utah, and Colorado), where interannual variation in greenup onset increased, but the interannual variation in temperature decreased (Fig. 3). In addition, the interannual variation of both spring phenology and temperature showed non-significant trends in the southeastern coastal regions (Fig. 3).

**Figure 5:**
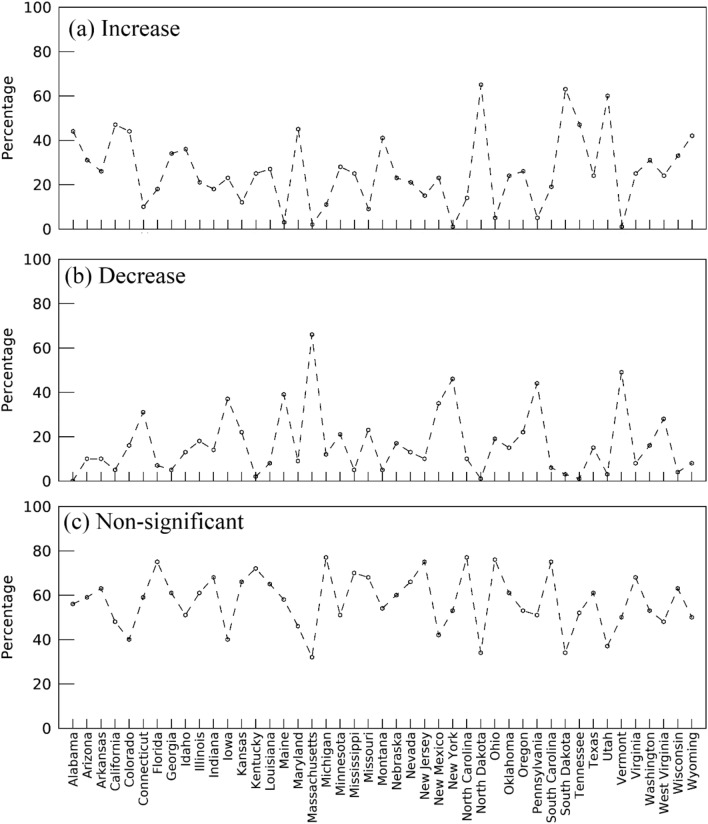
Percentage of pixels with (**a**) an increasing trend, (**b**) a decreasing trend, and (**c**) a non-significant trend (P > 0.1) of interannual variation in greenup onset across the CONUS.

The temporal trend in interannual variation in greenup onset varied across states (Fig. 5). The variation from 1982 to 2016 significantly increased in over 50% of pixels in the following states: North Dakota (65% of pixels), South Dakota (63%), Utah (60%) (Fig. 5a). However, it decreased in over 40% of pixels in Massachusetts (66% of pixels), Vermont (49%), New York (46%), and Pennsylvania (44%) (Fig. 5b). The states with the majority of pixels showing no significant trends are North Carolina (77% of pixels), Ohio (76%), South Carolina (75%), Kentucky (72%), Mississippi (70%), Virginia (68%), and Nevada (66%) (Fig. 5c).

### Extreme years of greenup onset and temperature

**Figure 6:**
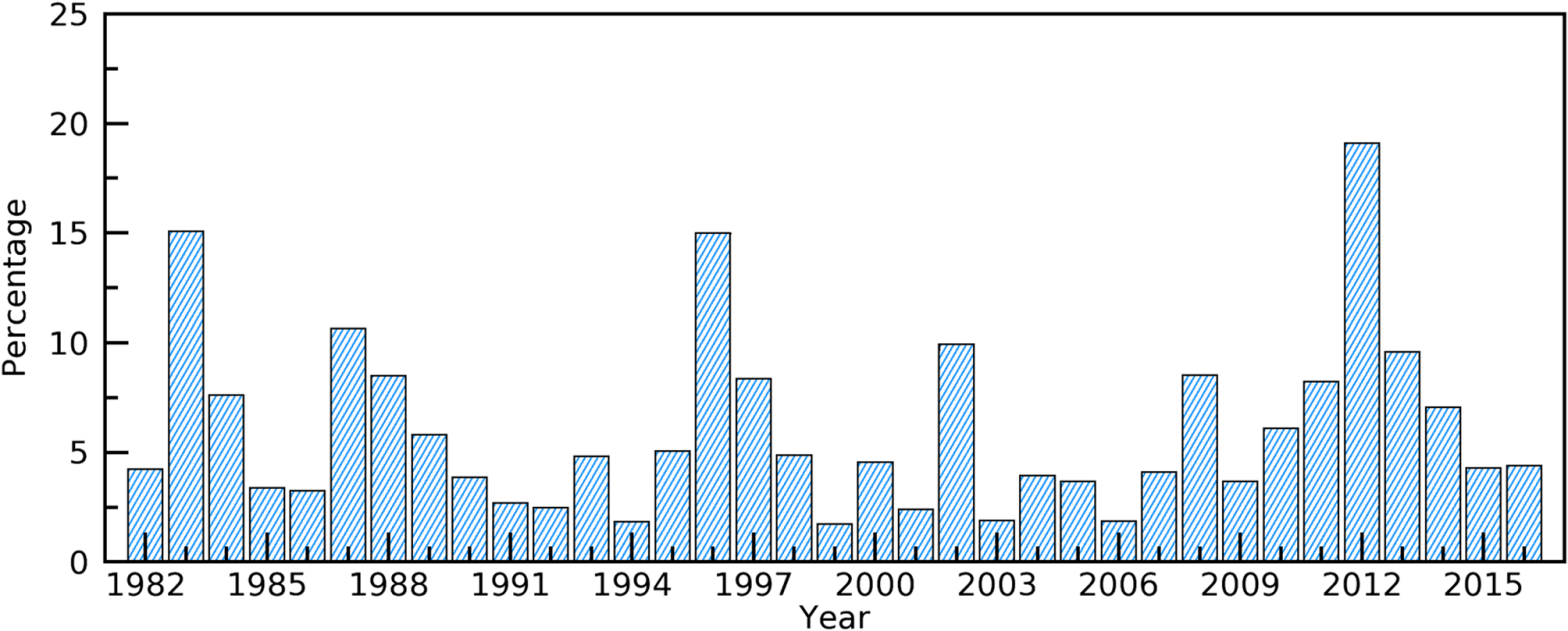
Percentage of pixels where greenup onset in a year was considered to be extreme across the CONUS.

Figure 6 shows the percentage of pixels where extreme greenup onset dates occurred across the CONUS. The extreme greenup onset percentage was largest in 2012, followed by 1983 and 1996. In these three years, the extreme greenup onset occurred in 19.1%, 15.1%, and 15.0% of the land across the CONUS, respectively. The percentages of pixels with extreme greenup onset in 1987 and 2002 were also distinctive: these years had percentages of 10.6% and 9.9%, respectively.

**Figure 7:**
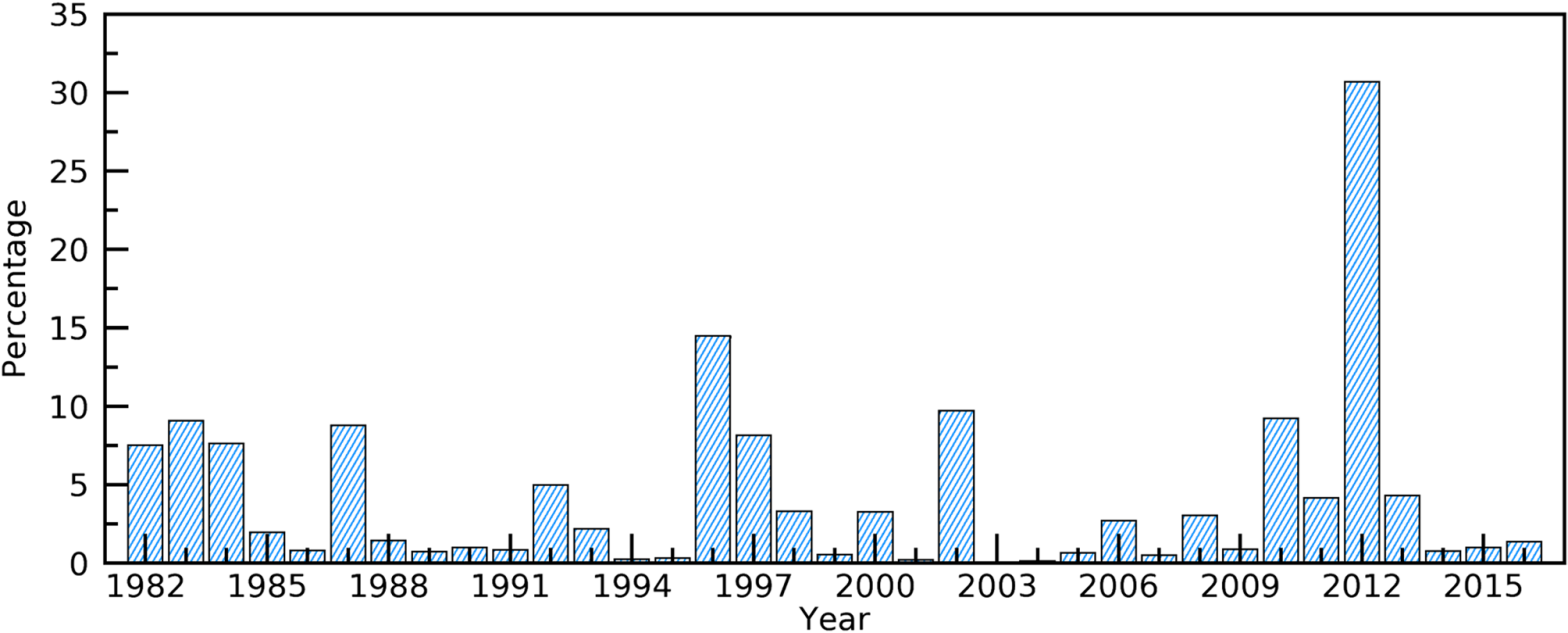
Percentage of pixels where spring temperature in a year was considered to be extreme across the CONUS.

The occurrences of extreme greenup onset corresponded well to extreme spring temperatures. Figure 7 shows the percentage of pixels where spring temperature was considered to be extremely hot or cool compared to a 35-year temperature mean value. The percentage of pixels with extreme spring temperatures was largest in 2012 (30.7%), followed by 1996 (14.5%), 2002 (9.7%), 1983 (9.1%) and 1987 (8.8%). Clearly, the years when large areas experienced extreme spring temperature were consistent with those that had an extreme greenup onset.

### Anomaly of greenup onset and temperature in three extreme years

**Figure 8:**
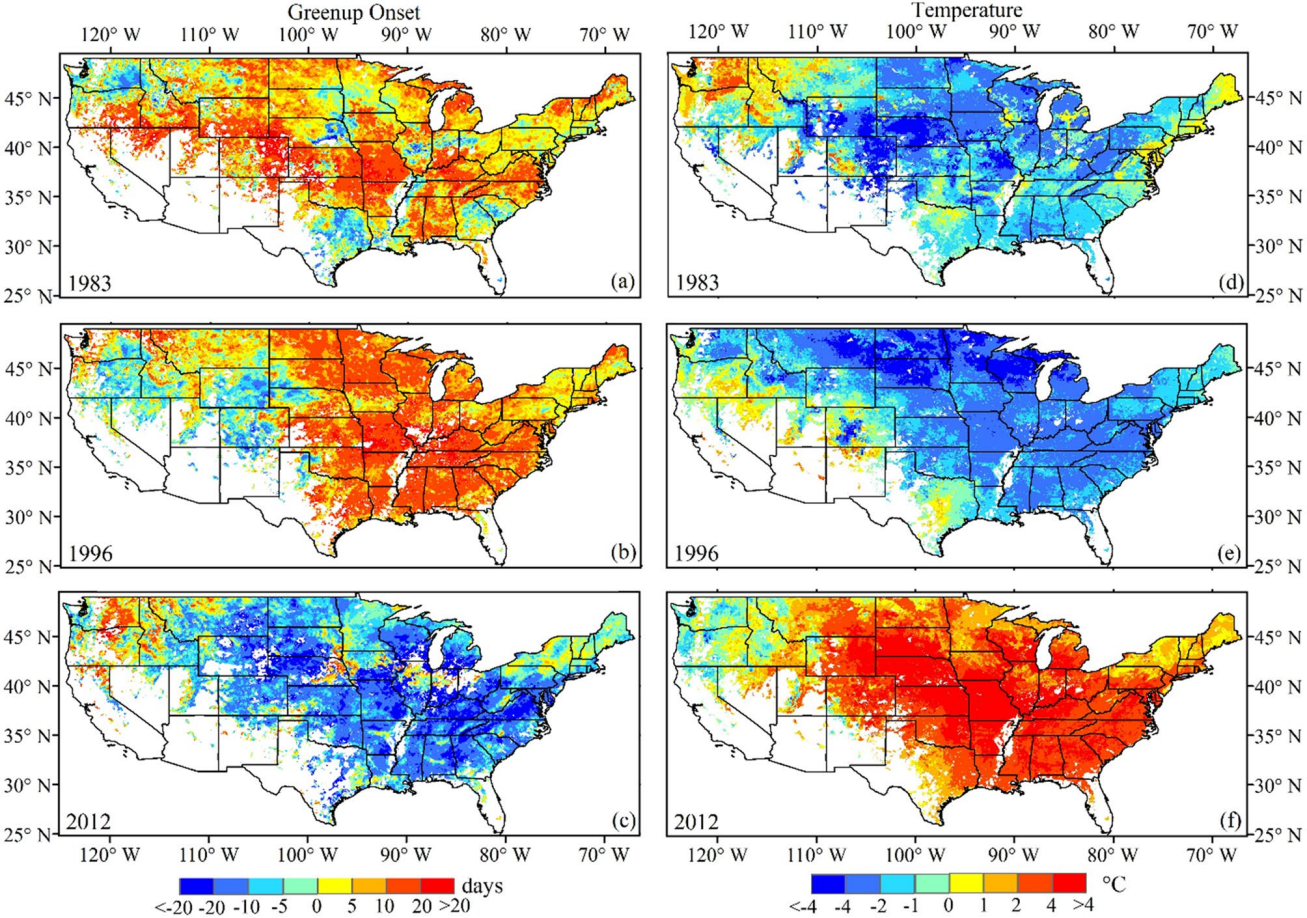
Spatial pattern of (**a**–**c**) greenup onset anomaly and (**d**–**f**) spring temperature anomaly in 1983, 1996, and 2012. The white color represents water or pixels where greenup onset was not continuously detected from 1982–2016.

Figure 8 displays the spatial pattern of greenup onset and spring temperature anomalies relative to the mean value from 1982 to 2016 in the three most extreme years (1983, 1996, and 2012). In 1983 and 1996, there were 82% and 81% of pixels showing later greenup onset, 36% and 47% of which had a greenup onset that occurred more than 10 days later than average, respectively (Fig. 8a,b). Interestingly, the spring temperature anomaly was below zero in 83% and 92% of pixels, respectively, in these two years (Fig. 8d,e). It is worth noting that there were 37% and 55% of pixels in 1983 and 1996, respectively, with a spring temperature anomaly below minus 2 °C. These pixel proportions in greenup onset and temperature anomaly were closely comparable. In contrast, in 2012, an earlier greenup onset occurred in most regions across the CONUS (81% of pixels), and higher spring temperatures occurred in 92% of pixels (Fig. 8c,f). Among them, greenup onset was more than 10 and 20 days early in 45% and 10% of pixels, respectively. Correspondingly, the positive anomaly of spring temperature was more than 2 °C and 4 °C in 64% and 28% of pixels, respectively.

**Figure 9:**
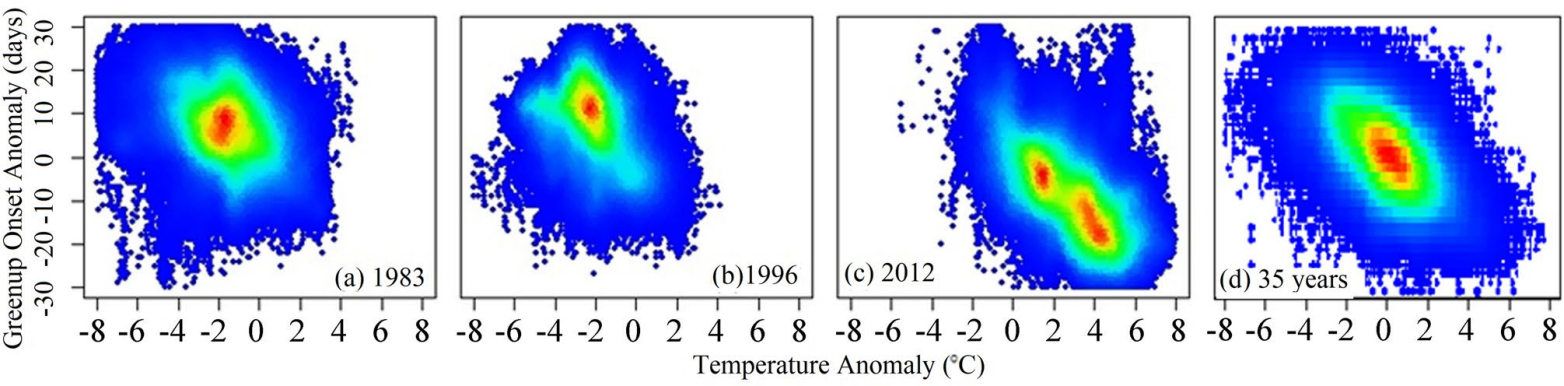
The density scatter plots between temperature anomaly and greenup onset anomaly in 1983, 1996, and 2012 and for 10% (to reduce the sample size) of randomly selected pixels from 1982–2016 (35 years). Note that the red color represents higher density and the blue color represents lower density.

Figure 9 displays the density scatter plots between temperature anomaly and greenup onset anomaly in 1983, 1996, and 2012 and from 1982–2016. Note that the sample size for the entire 35 years (1982–2016) was too large for anomaly analysis, so 10% of pixels were randomly selected in each year. In 1983, the highest pixel density (46.9%) mainly occurred in the -1/-3 °C temperature range and correspondingly, in the ~ 2–12 days (later, 48.5%) greenup onset anomaly range. This pattern was similar in 1996, although the greenup onset anomaly was relatively higher (~ 5–14 days). In 2012, in contrast, the highest density of the temperature anomaly occurred in two cluster areas (~ 0 °C –1 °C, 10.2% and ~ 2 °C–5 °C, 52.8%). Correspondingly, the highest density of greenup onset anomaly occurred in ~ -5–0 days (18.6%) and ~ -20– -10 days (39.7%). From 1982–2016, greenup onset anomaly was negatively correlated to temperature anomaly, although the anomalies ranged from -2 °C to 2 °C in temperature and from -10 to 10 days in greenup onset (Fig. 9d).

## Discussion

This is the first study to investigate the temporal trend in interannual variation of spring phenology across the entire CONUS, although the long-term spring phenology trend has been extensively investigated in previous studies. The trend in interannual phenology variations is independent of the commonly investigated temporal phenology trend. This is because the analysis of the temporal phenology trend focused on the change in direction of the trend after removing outliers caused by extreme climate events. However, the interannual phenology variation emphasizes the interannual phenology fluctuations resulting from climate variability. As the frequency and intensity of extreme climate events have rapidly increased and will continue to increase under changing climatic conditions^66,67^, investigating the trend of interannual phenological variation becomes more critical than investigating the regular long-term trend in phenological timings, because large interannual variability could have more significant impacts on ecosystems.

The increasing interannual variations in greenup onset were found in the standard deviation of greenup onset at decadal scales. Our results showed the largest variation occurred in the 2010s compared with variations in the 1980s, 1990s, and 2000s. Trend analysis of the five-year centered moving standard deviations further demonstrated the increases of interannual variations in greenup onset from 1982 to 2016. The interannual variations increased in 62% of pixels (29% of pixels with P < 0.1) across the CONUS. Nevertheless, the decreasing trend in interannual variations only appeared in 38% of pixels (15% of pixels with P < 0.1). This indicates that long-term spring phenology experienced increasing interannual variability.

The interannual variation in greenup onset could be to a great extent explained by the variation in spring temperature. Our results showed that interannual variation of both greenup onset and spring temperature revealed consistent trends in 60% of pixels, 44% of which showed an increasing trend, which suggests that both phenology and temperature show similar trends. Our analysis further revealed that the interannual variation in phenology on a decadal scale was generally comparable with the temperature variation, particularly in the 2010s (the figure was not presented for conciseness). These findings were supported by the recent report that more than half of interannual variation in greenup onset was explained by the variation in spring temperature^68^. However, there were only 14% of pixels showing consistently significant trends in both phenology and temperature. The small percentage of pixels with significant trends is quite common in the field of phenology and climate change, as reported in many previous studies^15,16^, because the significance level may change with the time period of the data and interannual variations. Overall, the spatial pattern of phenological trends follows the variation in spring temperature. However, the spatial pattern of interannual temperature variation is likely influenced by complex interactions of several forcing factors at different temporal scales, such as the Pacific/North American circulation pattern (days to weeks), the El Niño and La Niña Southern Oscillations (1–2 years), and the Pacific Decadal Oscillation (20–30 years)^69,70^.

The decreasing interannual variations in both greenup onset and spring temperature mainly occurred in the forested area spread over the northeastern CONUS. This finding is supported by previous studies showing that the smallest interannual variation occurred in forests in North America and China compared to other biomes, such as grasslands and croplands^71,72^. The decreasing variation suggests that forest ecosystems were relatively stable during the past several decades. This pattern suggests that the variation of forest ecosystem greenup is mainly driven by temperature, although the impacts of ecological factors light availability, species and tree size^73,74^ need to be investigated in the future. However, opposite trends in interannual variations were found for greenup onset and spring temperature; this mainly occurred in the Rocky Mountain area. This is likely due to the effects of snow conditions such as very likely increasing variability and decreasing trend in snow fall in last several decades^75^ and topography on the arrival of greenup onset^58^, and it is also likely related to the spatial mismatch between LSP data (~ 5 km) and temperature data (32 km) in mountainous ecosystems.

The temporal trend in interannual variation in greenup onset also differed greatly across states. Large increasing variation occurred in most states over the central CONUS, where croplands were distributed. For example, about 70% of pixels showed a significant increasing interannual variation in greenup onset in South Dakota and North Dakota. This pattern was consistent with the increasing interannual variation of spring temperature. Increasing interannual variations in crop phenology are a double-edged sword in regulating crop yield. On the one hand, famers could take advantage of earlier planting and sow longer‐season cultivars^76^. On the other hand, large interannual variations could result in crop yield loss due to the lag in the adaptation of crops to extreme climate events^77,78^. Therefore, the increasing interannual variability in crop spring phenology could lead to increases in yield variability^79,80^, which in turn would challenge food security in the United States. Knowledge of phenological variation at a state level and in local regions could assist with the assessment of climate impacts for various audiences^60^.

A large anomaly in greenup onset was considered to be extreme, and it could be found in local regions with variable sizes in each individual year from 1982 to 2016. However, an extreme greenup onset appeared in 1983, 1996, and 2012 in most regions of the CONUS. This extreme spring phenology is a response to extreme spring temperatures. Due to the fact that the temperature is a major driver of spring phenology^81,82^, an extremely hot spring could advance spring phenology to a great extent, and vice versa. This result was supported by several previous studies that showed that anomalies of spring phenology were primarily driven by temperature anomalies across the CONUS^38,83,84^.

The results in this study clearly demonstrated that the extreme delays of greenup onset in 1983 and 1996 were a response to extremely cool temperatures, and the extremely early onset of greenup in 2012 was a response to the extremely warm spring across the CONUS. Detecting extreme years in spring phenology could also improve our understanding of long-term phenology trends, because individual extreme values could have a substantial influence on the statistical significance and even the direction of temporal trends. During the next few decades, it is most likely that large interannual variations in spring phenology could frequently occur and that increasing interannual variation could substantially reshape the ecological community and further challenge ecosystem sustainability^85^. This is because (1) climate warming will continue in the near future because the emission of greenhouse gases from human activities continues to increase according to the Intergovernmental Panel on Climate Change’s (IPCC) fifth assessment report ^86–88^, and (2) undergoing global warming would increase the probability of extreme climate events^89,90^ and could further exacerbate the influence of weather and climate extremes on vegetation growth.

In addition to temperature, an extreme spring phenology could also be caused by other factors. It could be the result of extreme rainy seasons or drought episodes in the arid or semiarid regions, where soil moisture plays an important role in addition to temperature^61,91^. Moreover, an extreme spring phenology could be associated with disturbances, such as fires and deforestation^92,93^. In this study, we mainly focused on temperature-controlled ecosystems, which cover most regions in the CONUS. Due to the fact that spring vegetation phenology in the western CONUS with much higher interannual variation could be driven by the seasonality of rainfall or snow fall^83,94^, phenological interannual variations were not investigated in this study. It should be noted that detecting phenological events is very challenging in semiarid regions, where seasonal vegetation dynamics are complex, so that most currently available satellite-derived phenology products exclude phenology detections in the western CONUS^95^. Some studies found strong relationships between vegetation and precipitation variations^61^, but other studies showed that a large interannual variation in precipitation caused by heavy rainfall was not related to the variation in vegetation^96,97^. It is likely that precipitation variation could have a more complex influence on phenology variation; this requires further investigation. In addition, the percentages of pixels with extreme events of phenology and temperature were not exactly the same, which could be related to the determination of extreme events and the spatial resolution of the data sets (32 km in temperature and 5 km in phenology). The determination of extreme events using the same mathematical methods (two standard deviations) in this study could be improved in the future using different criteria.

Finally, analyzing interannual variations in spring phenology could be influenced by the selection of the time period (or window size). The selected window size could impact the magnitude values of interannual variations (defined as the standard deviation within the window in this study), the sample number of the time series, and the significance level of long-term trends in statistical analysis. However, the window size would have a very limited influence on the direction of the temporal trend in interannual variations, which was tested in our analysis. In addition, the decadal interannual variation in greenup onset was calculated using data from 1982–1989 (8 years), 1990–1999 (10 years), 2000–2009 (10 years), and 2010–2016 (7 years) to represent the 1980s, 1990s, 2000s, and 2010s. This difference in the number of years in each period could cause some inconsistences in the interdecadal comparison. Satellite observations made during the period of 2017–2019 will be included to investigate the interannual variations in the 2010s in the near future.

In summary, the results from this study suggest that interannual variation in greenup onset could continuously increase because of the increase of climate variation in next few decades. Increased climate interannual variations could lead to a high frequency of extreme phenological events.
